# Is entrepreneurship a key factor in the development of European countries? A proposal for an innovation readiness environment (IRE) index

**DOI:** 10.12688/openreseurope.16813.1

**Published:** 2024-01-08

**Authors:** Elisa Fabbro, Yuliia Kyrdoda, Salvatore Dore, Giacomo Marzi, Giuseppe Borruso, Silvia Battino, Giovanni Cristiano Piani, Donata Vianelli

**Affiliations:** 1 Internationalization Staff Unit, Institutional Services Area, University of Trieste, Trieste, Friuli-Venezia Giulia, Italy; 2 Department of Economics, Business, Mathematics and Statistics, University of Trieste, Trieste, Friuli-Venezia Giulia, Italy; 3 Technology Transfer Office, University of Trieste, Trieste, Friuli-Venezia Giulia, Italy; 4 IMT School for Advanced Studies Lucca, Lucca, Tuscany, Italy; 5 Department of Economics and Business, University of Sassari, Sassari, Sardinia, Italy; 6 Communication and External Relations, University of Trieste, Trieste, Friuli-Venezia Giulia, Italy

**Keywords:** Research and Development, Innovation, Entrepreneurship, Innovation Readiness Environment Index, Tertiary Education, Patent Application, Spatial Analysis

## Abstract

This study investigates the complex interplay among innovation, research and development (R&D), and entrepreneurship within the context of European nations. The focus of the study is also on the contributory role of tertiary educational institutions in nurturing entrepreneurial activities. To deepen the understanding of these multifaceted relationships and their subsequent impact on regional economies, the research introduces a novel metric termed the Innovation Readiness Environment (IRE) index. This index combines various indicators such as R&D expenditure, patenting rates, firm size, and educational levels, thereby providing a framework for evaluating the innovative capabilities and entrepreneurial success of firms in a given region. Utilization of this index offers policymakers and stakeholders a nuanced understanding of the regional innovation ecosystem, facilitating the identification of both strengths and deficiencies. This, in turn, enables the formulation of targeted policy interventions to enhance both innovation and entrepreneurship. One salient conclusion drawn from this study is the pivotal role of tertiary education in catalysing entrepreneurial ventures. The findings posit that higher levels of entrepreneurial education significantly supplement an individual's likelihood of entrepreneurial success by imparting the requisite skills and knowledge indispensable in a competitive business milieu. Higher education institutions, by fostering an environment conducive to innovation, emerge as critical agents in the cultivation of entrepreneurial acumen and the stimulation of economic expansion. The study further incorporates a spatial analytical framework to elucidate the regional specificities of innovation at the pan-European scale.

## Plain language summary

The present research examines the interconnected roles of innovation, research and development, and entrepreneurship in European countries, particularly emphasising how higher education contributes to entrepreneurial activity. Through the introduction of the Innovation Readiness Environment (IRE) index, the study provides a structured way to measure various aspects like research spending, patent rates, and educational levels in a region.

The IRE index is intended to guide policymakers and other key stakeholders. The index is a tool for comprehensively assessing regional strengths and weaknesses in innovation and entrepreneurship. Furthermore, the study underscores the crucial role of higher education in fostering entrepreneurial skills, thereby enhancing the probability of entrepreneurial success. As a result, it appears that universities are not just places for acquiring knowledge; they serve as incubators for new ideas and start-ups, contributing to broader economic growth.

## 1. Introduction

In the context of the 2030 Agenda for Sustainable Development (SDG), ( https://sdgs.un.org/2030agenda), entrepreneurship plays a pivotal role in enhancing society's quality of life, including for disadvantaged groups, as it contributes to building resilient infrastructure, promoting inclusive and sustainable industrialization, and fostering innovation. Entrepreneurship is closely linked to SDGs 4 and 8, reviewed in 2019. SDG target 4.4 aims to significantly increase the number of youth and adults with relevant skills, including technical and vocational skills, for employment, decent jobs, and entrepreneurship. Simultaneously, SDG target 8.3 supports development-oriented policies that promote productive activities, decent job creation, entrepreneurship, creativity, and innovation while encouraging the formalization and growth of micro-, small-, and medium-sized enterprises (MSMEs) as critical agents for and beneficiaries of inclusive development through access to financial services.

Entrepreneurs with a strong commitment to sustainable development contribute to achieving almost all SDGs, as they create businesses that support employment, alleviate poverty, and enable decent work and economic growth. Furthermore, entrepreneurship benefits efforts to reduce hunger, promote good health and well-being, achieve affordable and clean energy, and strengthen industries. Entrepreneurship can be the driving force behind transforming our world and overcoming diverse global challenges ( Apostolopoulos, 2018).

Recognizing the importance of entrepreneurs and entrepreneurship, the European Commission has launched the "Entrepreneurship 2020 Action Plan," which aims to unleash Europe's entrepreneurial potential, eliminate existing obstacles, and modernize the culture of entrepreneurship in Europe. These goals can be achievable through fostering an entrepreneurial mindset through entrepreneurship education in higher education institutions. Universities' teaching activities shape students' entrepreneurial orientations and competencies. Recent research suggests that university entrepreneurship programs may not increase the rate of entrepreneurship, but they do help students to better identify their entrepreneurial potential and improve the performance of their start-ups, leading to higher success rates, improved employability, and better management skills. To foster entrepreneurial spirit, create prosperity and well-being, and facilitate European system growth, it is crucial to consider regional differences and analyse regional innovation indicators. This necessitates understanding each region's trajectory through tools like the Smart Specialization Strategy (S3) and Smart Specialization Strategies for Sustainable and Inclusive Growth (S4). To better comprehend regional situations, we propose an "ad hoc" index reflecting the entrepreneurial tendencies of specific regional territories.

The remainder of the paper is organized as follows: Following the introduction in Paragraph 1, Paragraph 2 elucidates the theoretical underpinnings of the IRE index. Paragraph 3 delineates the methodology for selecting the appropriate variables and explicates the steps involved in the creation of the IRE index, whereas Paragraph 4 offers a discussion of the results. Finally, Paragraph 5 provides the concluding remarks.

## 2. Theoretical background

The Entrepreneurship 2020 Action Plan is a comprehensive strategy for catalysing and disseminating the culture of entrepreneurship in Europe through the transformation of entrepreneurial universities and the nurturing of entrepreneurial spirit. The plan is structured around three key pillars, namely, entrepreneurial education and training, fostering an environment in which entrepreneurs can flourish and grow, and developing role models and engaging specific groups with untapped entrepreneurial potential or those not reached by traditional business support methods.

The first pillar emphasizes the importance of expanding and enhancing entrepreneurial education and training, which is considered one of the most significant investments Europe can make. The acquisition and implementation of entrepreneurship skills have also been highlighted within the framework of EU cooperation in the field of education ( Jenner, 2012). Overall, investments in entrepreneurial education are efficient tools for raising public awareness of entrepreneurs and supporting underrepresented groups among entrepreneurs. Only if a large number of Europeans perceive an entrepreneurial career as a rewarding and attractive option will entrepreneurial activity thrive in Europe in the long term.

However, Benneworth and Osborne (2015) argued that entrepreneurship education has not yet reached its full potential, partly due to poor integration with other knowledge activities at universities. The authors suggest that future research on university entrepreneurship education should focus on how entrepreneurial activities align with universities' core knowledge, providing a more coherent understanding of universities' contributions to fostering entrepreneurial attitudes.

The global configuration of knowledge and technology is one of the most critical factors influencing the pursuit of internationalization and the rise of the global economy ( Posselt *et al.,* 2019). The rapid evolution of technology and market demands necessitates the development of an entrepreneurial spirit, digital literacy, and innovative learning and teaching methods. In this context, entrepreneurial universities must adapt by cultivating leadership, navigating complexity, adopting a lifelong learning approach, and transforming failure into success ( Gibb *et al.,* 2013).

These four essential features of entrepreneurs and entrepreneurial universities play a crucial role in promoting entrepreneurship in Europe. By focusing on these core values and principles, the Entrepreneurship 2020 Action Plan aims to revolutionize the landscape of entrepreneurship and support Europe's long-term economic growth and development.

In the following sub-sections, we present an exploration of the key indicators that were scrutinized during the development of the IRE index. The aim is to inform their respective significance and the resultant impact they exert on the innovation readiness of a given region.

### 2.1 The role of tertiary education on the population aged 25–34

Education is essential both at the individual level, as it is key to navigating the world, and at the national level, as it contributes to a country's economic and societal development. Recent research has emphasized the critical role of education as a supply-side factor in the context of entrepreneurship and innovation, particularly in tertiary education, also known as post-secondary education or advanced studies ( Crecente-Romero *et al.,* 2018; van Praag & van Stel, 2013).

Tertiary education is shaped by government policies and is seen as an effective instrument for advancing human and national development. According to Peña-Vinces & Audretsch (2021), its primary aim is to enhance the educational and professional levels of employees across various specializations. This, in turn, increases the number of highly skilled workers, which boosts company performance and, consequently, the country's economy.

Furthermore, research conducted by Millán *et al.* (2014) revealed that education levels are positively related to entrepreneurial success, as highly educated individuals contribute to a company's prosperity either by running their own businesses or by providing their skills and expertise. Similarly, Jiménez *et al.* (2015) argued that the impact of tertiary education is twofold. On one hand, it increases formal entrepreneurship due to higher self-confidence and lower perceived risk, while on the other hand, it reduces informal business activity. The negative relationship stems from an increased awareness of and sensitivity to the potential negative consequences of certain activities. In parallel, Barreneche García (2014) found that the more individuals are involved in tertiary education, the greater the positive entrepreneurial dynamism.

Taking together the evidence from the literature, the indicator of tertiary education is seen as a vital factor for fostering entrepreneurial activities. As a result, we considered it as a general measure of the supply of advanced skills across various industries and sectors.

**Figure 1.**
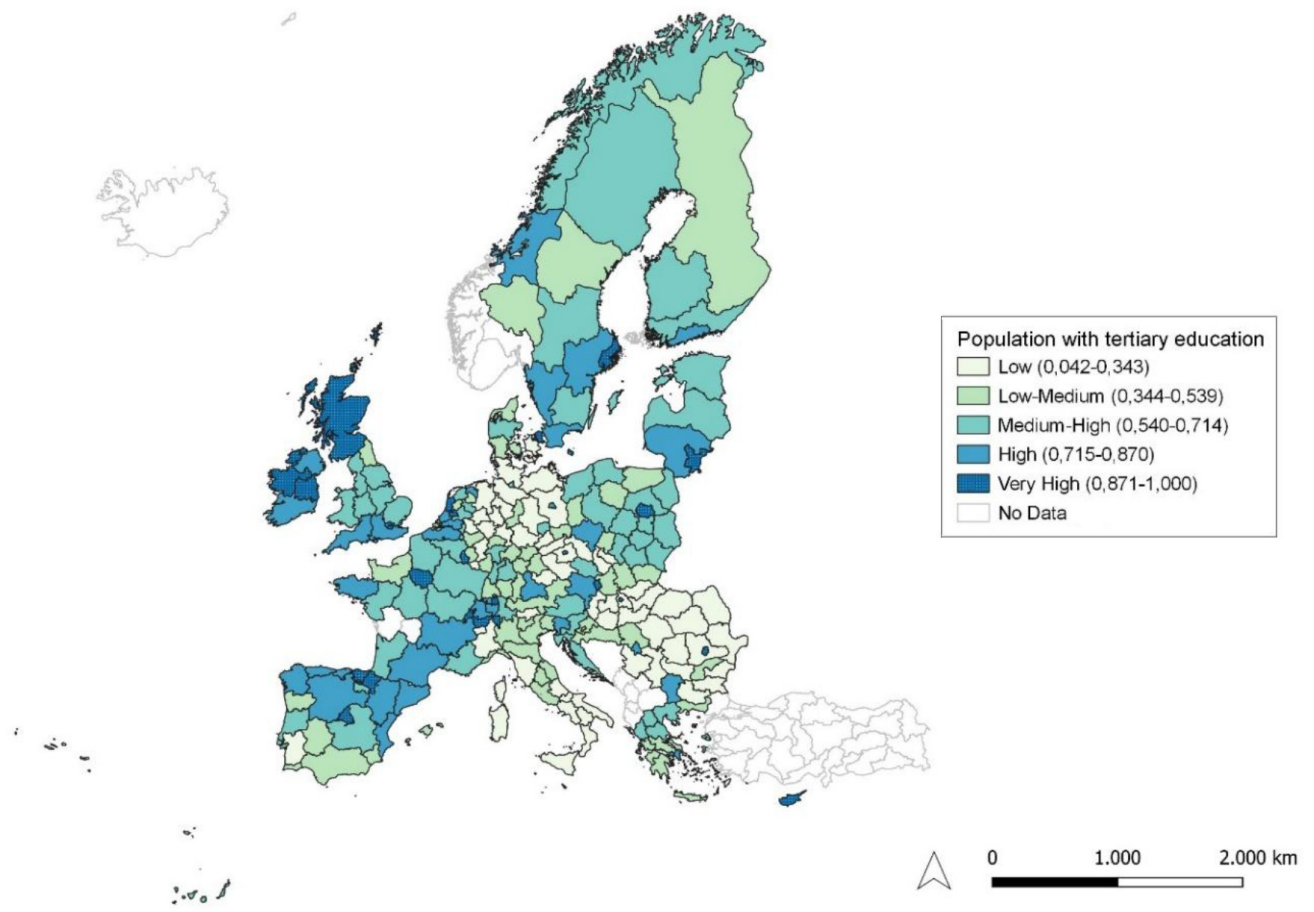
Percentage population aged 25–34 having completed tertiary education. Source: elaboration from GISCO – Eurostat data; Data as in Table 1. Map by Silvia Battino.

**Table 1.** The selected indicators and data sources.

Indicator	Numerator	Denominator	Data sources	Data availability
*Percentage population * *aged 25–34 having * *completed tertiary * *education*	Number of persons in age class with some form of post-secondary education	The reference population is all age classes between 25- and 34-years inclusive	Eurostat, regional statistics, Regional innovation scoreboard 2021	NUTS 2: 2012 – 2019
*Percentage population * *aged 25–64 participating * *in lifelong learning*	Number of persons in private households aged between 25 and 64 years who have participated in the four weeks preceding the interview, in any education or training, whether or not relevant to the respondent’s current or possible future job	Total population aged between 25 and 64 years	Eurostat, regional statistics, Regional innovation scoreboard 2021	NUTS 2: 2012 – 2019
*R&D expenditure in the * *business sector*	All R&D expenditures in the government sector (GOVERD) and the higher education sector (HERD)	Regional Gross Domestic Product	Eurostat, regional statistics, Regional innovation scoreboard 2021	NUTS 2: 2011 – 2018
*Innovative SMEs * *collaborating with others * *as percentage of SMEs*	Number of SMEs with innovation co-operation activities. Firms with co-operation activities are those that have had any co-operation agreements on innovation activities with other enterprises or institutions.	Total number of SMEs	Community Innovation Survey: Eurostat and National Statistical Offices	NUTS 1 and 2 for different countries for CIS 2012, CIS 2014, CIS 2016, CIS 2018
*PCT patent applications * *per billion regional GDP*	Number of patents applied for at the European Patent Office (EPO), by year of filing.	Gross Domestic Product in Purchasing Power Standard	Numerator: OECD, REGPAT. Denominator: Eurostat	NUTS 2: two-year averages for 2012 - 2019
*Sales of new-to-market * *and new-to-firm * *innovations in SMEs as * *percentage of turnover*	Sum of total turnover of new or significantly improved products for SMEs	Total turnover for SMEs	Community Innovation Survey: Eurostat and National Statistical Offices	NUTS 1 and 2 for different countries for CIS 2012, CIS 2014, CIS 2016, CIS 2018

Sources: authors’ elaborations based on data as listed in the table.

### 2.2 The role of lifelong Learning on innovation activities

According to the European Commission's definition, lifelong learning encompasses "all learning activity undertaken throughout life, with the aim of improving knowledge, skills, and competencies within a personal, civic, social, and/or employment-related perspective." In other words, it refers to all formal, non-formal, and informal learning. Furthermore, Jarvis (2007) expanded the concept of learning by specifying two types-vocational and non-vocational-and emphasized that learning covers any opportunity to acquire new knowledge, skills, attitudes, values, emotions, beliefs, and senses through social institutions or any process.

The concept of lifelong learning can be viewed from three perspectives: individual lifelong learning, learning organizations, and learning societies ( Tight, 1998). These dimensions underscore the value of learning for development at the individual, company, and national levels. For example, Coelli & Tabasso (2019) study found that engaging in education tends to improve labour market performance and, specifically, employee outcomes such as working hours or wage rates. Moreover, lifelong learning benefits society by potentially increasing company output and tax revenues. In the context of learning organizations, McKelvey (1998) emphasized that the connection between learning and innovation enables companies to overcome challenges through their ability to learn and adapt.

For further analysis, the lifelong learning indicator is essential, as it strongly correlates with innovation activities (such as the development of artificial intelligence and nanotechnologies). Overall, lifelong learning, including both formal and informal education, enables the enhancement of knowledge, skills, and competencies.

**Figure 2.**
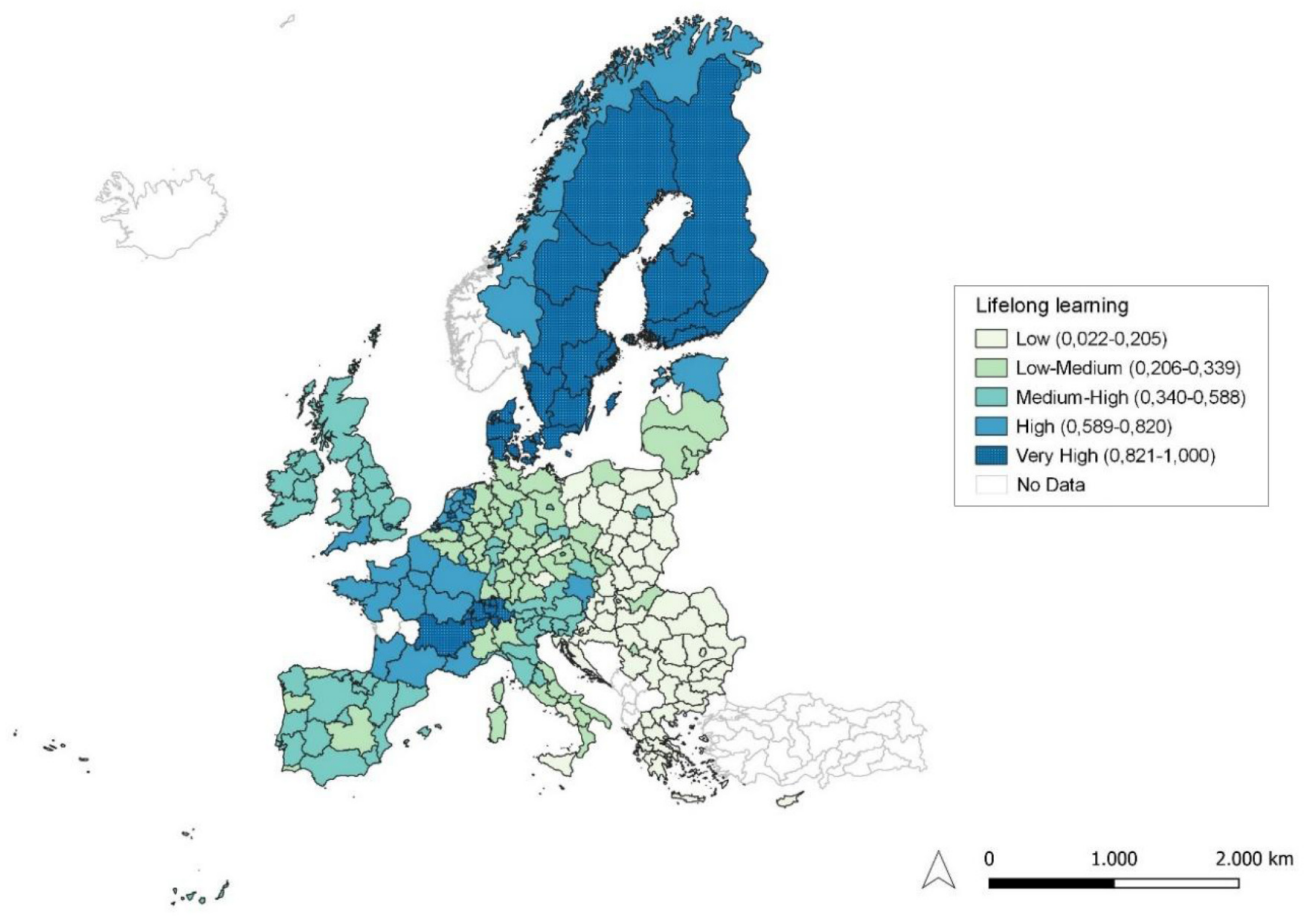
Lifelong learning. Source: elaboration from GISCO – Eurostat data; Data as in Table 1. Map by Silvia Battino.

### 2.3 The role of R&D expenditure in the entrepreneurial ecosystem

Research and Experimental Development (R&D) encompasses innovative and structured actions aimed at obtaining new knowledge and enhancing existing knowledge (Eurostat). In recent years, extensive literature has focused on the relationship between R&D expenditures and firm performance. This interest stems from Schumpeter's theory, which posits that companies attempt to foster innovation in order to bolster their competitive advantages, potentially leading to higher profitability, productivity, and even market monopolies ( Capasso *et al.,* 2015). Today, interest in innovation continues to grow due to the rapid development of technologies and the availability of highly skilled labour. Another contributing factor is the opportunity to expand activities in foreign markets, causing R&D to become increasingly supply-driven as companies adopt a more global orientation ( Siedschlag *et al.,* 2013).

However, R&D investments can be challenging, as the positive impact of R&D on a company's innovative performance may increase up to a certain point, after which further R&D spending may lead to diminishing performance ( Berchicci, 2013). Nonetheless, most scholars emphasize the beneficial role of R&D, particularly for a firm's productivity growth ( Cin *et al.,* 2017; Kancs & Siliverstovs, 2016; Wakelin, 2001), marketing performance ( Sharma *et al.,* 2016), employment rates ( Di Cintio *et al.,* 2017), and overall future performance ( Ruiqi *et al.,* 2017). Consequently, company growth tends to improve the economic situation within a country. For instance, research found that R&D expenditures, along with educational factors, are most efficient in increasing GDP per capita. However, the analysis also revealed that the impact of R&D varies among regions; it is significant only in the most developed regions of Europe, while education is relevant in all cases ( Sterlacchini, 2008).

R&D expenditures are considered one of the most influential drivers of economic growth at both the company and national levels. Additionally, they are crucial for transitioning to a knowledge-based economy, as they advance production technologies, which, in turn, lead to economic growth. For this reason, we included this measure in our analysis, as it may provide a more comprehensive view of the degree of innovativeness in European regions.

**Figure 3.**
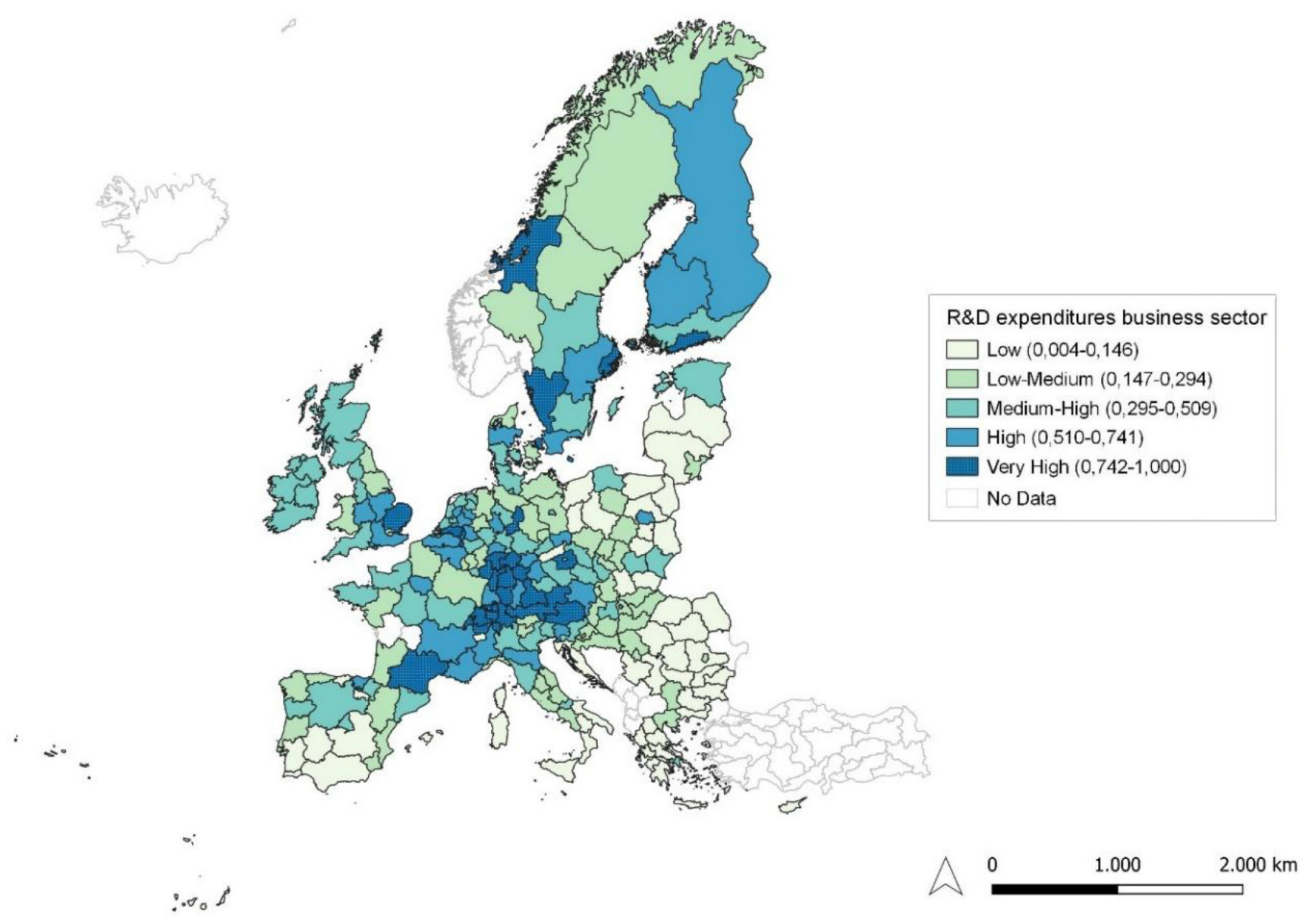
R&D expenditure in the business sector. Source: elaboration from GISCO – Eurostat data; Data as in Table 1. Map by Silvia Battino.

### 2.4 The role of innovative SMEs and their collaboration activities

Due to their size, small and medium enterprises (SMEs) are often more vulnerable to challenges in the business environment. Nevertheless, the majority of academic evidence suggests that innovations can be effective solutions for enhancing SMEs' performance. For example, Hall *et al.* (2009) investigated the relationship between innovations and firm productivity, discovering that R&D intensity and investments in equipment are positively related to performance outcomes. Moreover, Laforet (2013) found that SMEs with an innovation orientation tend to be more successful in the market, as they can quickly respond to market demand with better quality products or services.

In addition to improving performance domestically, innovations can also help SMEs expand their international activities. By implementing organizational and product innovations, companies can bolster their marketing innovations. Integrating these innovations with technological advancements enables SMEs to increase exports ( Bodlaj *et al.,* 2020). Meanwhile, the analysis of Saridakis *et al.* (2019) observed that innovative SMEs are more likely to extend their activities overseas than their non-innovative counterparts. Furthermore, the impact of innovation on internationalization varies among SMEs depending on the nature of the innovation and the degree of novelty. Similarly, Rosenbusch *et al.* (2011) identified additional factors affecting innovation outcomes, such as the age of the company and the cultural context.

Measuring innovative activities in SMEs advances research by revealing current market trends in terms of SMEs' adoption of open innovations within the European context. This indicator specifically focuses on SMEs that implement, promote, and collaborate with educational organizations on innovative initiatives. More precisely, it measures the flow of knowledge between public research institutions and firms, as well as between firms themselves.

**Figure 4.**
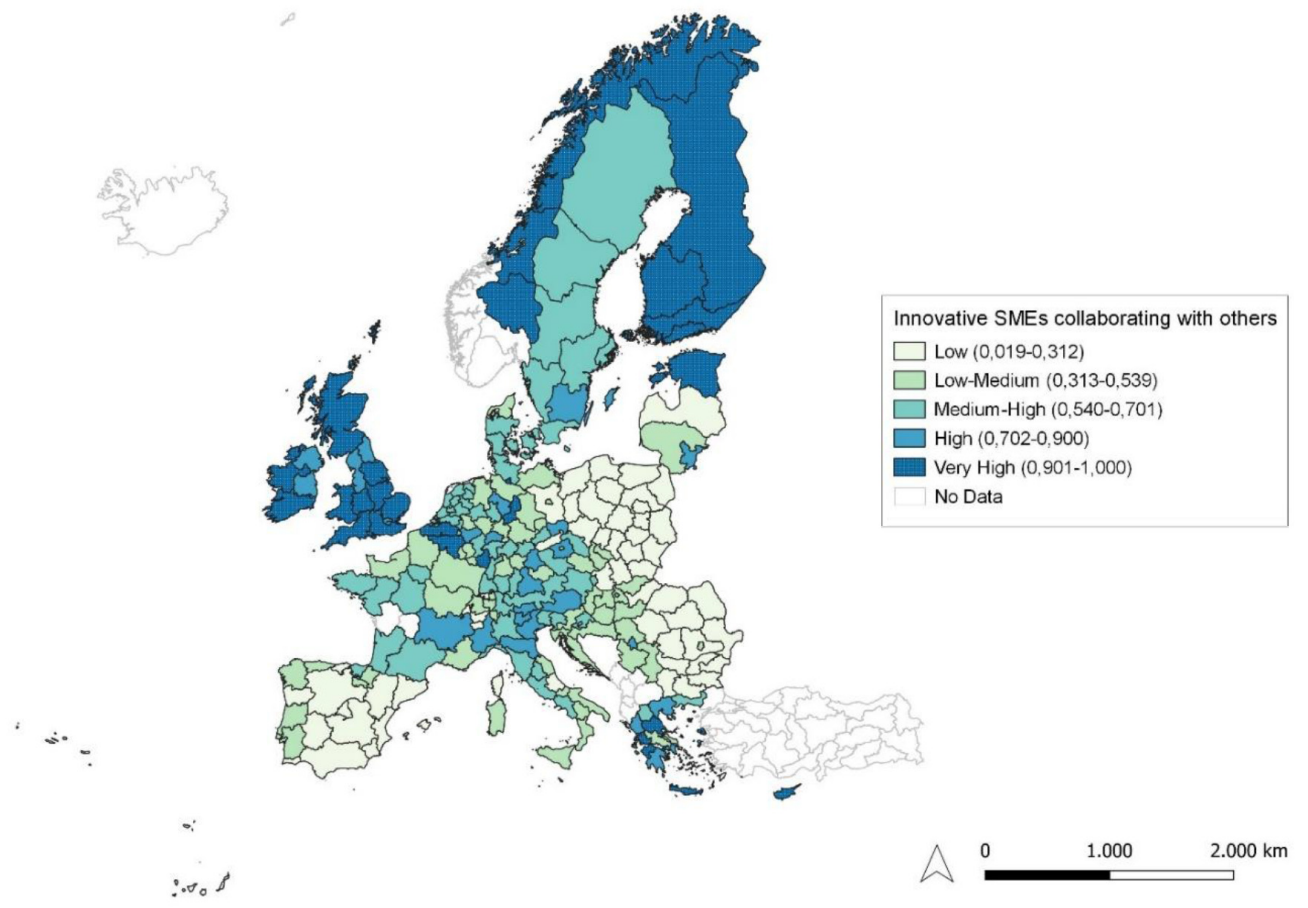
Innovative SMEs collaborating with others as a percentage of SMEs. Source: elaboration from GISCO – Eurostat data; Data as in Table 1. Map by Silvia Battino.

### 2.5 PCT patent applications

The context of innovation is closely related to patent applications, as they enable companies to distinguish their inventions overseas and protect intellectual property rights ( Ervits & Zmuda, 2018). Patent applications are commonly used as indicators of innovation output within academic literature. For instance, Bronzini & Piselli (2016) assessed firm innovation through patenting activity, finding that the implementation of regional subsidy R&D programs led to an increase in patent applications, which in turn positively impacted a company's innovation activities. Meanwhile, their analysis revealed company size as a distinguishing factor, with SMEs tending to exhibit higher intensity and likelihood of patenting than large firms. In contrast, Athreye *et al.* (2021) argued that larger companies can produce more patentable innovations due to existing cost barriers. Similarly, the study of Arundel & Kabla (1998) established a link between firm size and patent propensity rates. Furthermore, patents were identified as tools for protecting products and processes from being copied by competitors in the market.

Another line of research has focused on exploring the role of patent applications at the regional or national level. From a regional perspective, Lin *et al.* (2022) developed a two-stage model of the value-creation process within a regional innovation system, taking into account various patent statuses. Their findings revealed that invention patents play a crucial role in the overall performance of regional innovation development, as increasing patent applications lead to higher regional innovation scores. In another study, Whitacre *et al.* (2019) examined methods for measuring business innovation, creating an innovation index that allowed for the analysis of the relationship between innovation and firm- and regional-level outcomes. As a result, the research determined a positive impact of innovations on both firm and regional outcomes, with companies benefiting from increased employee wages and market share. Simultaneously, at the regional level, innovations influenced household income, the percentage of employees in the creative class, and poverty levels. In a broader view, Ervits & Zmuda (2018) conducted a cross-country analysis focusing on the impact of corruption and the business climate on patenting activity. It is noteworthy that countries with higher scores in institutional environments tend to exhibit greater incentives for patent applications. Consequently, companies, particularly SMEs that face a lack of ownership advantages and other resources, may benefit from such environments.

Patent applications can be seen as an indicator of a firm's ability to develop new products, which in turn leads to increased competitive advantages. As such, it is a critical measure of a company's innovative activities. More specifically, we considered this indicator as the number of patent applications per year within the context of European regions.

**Figure 5.**
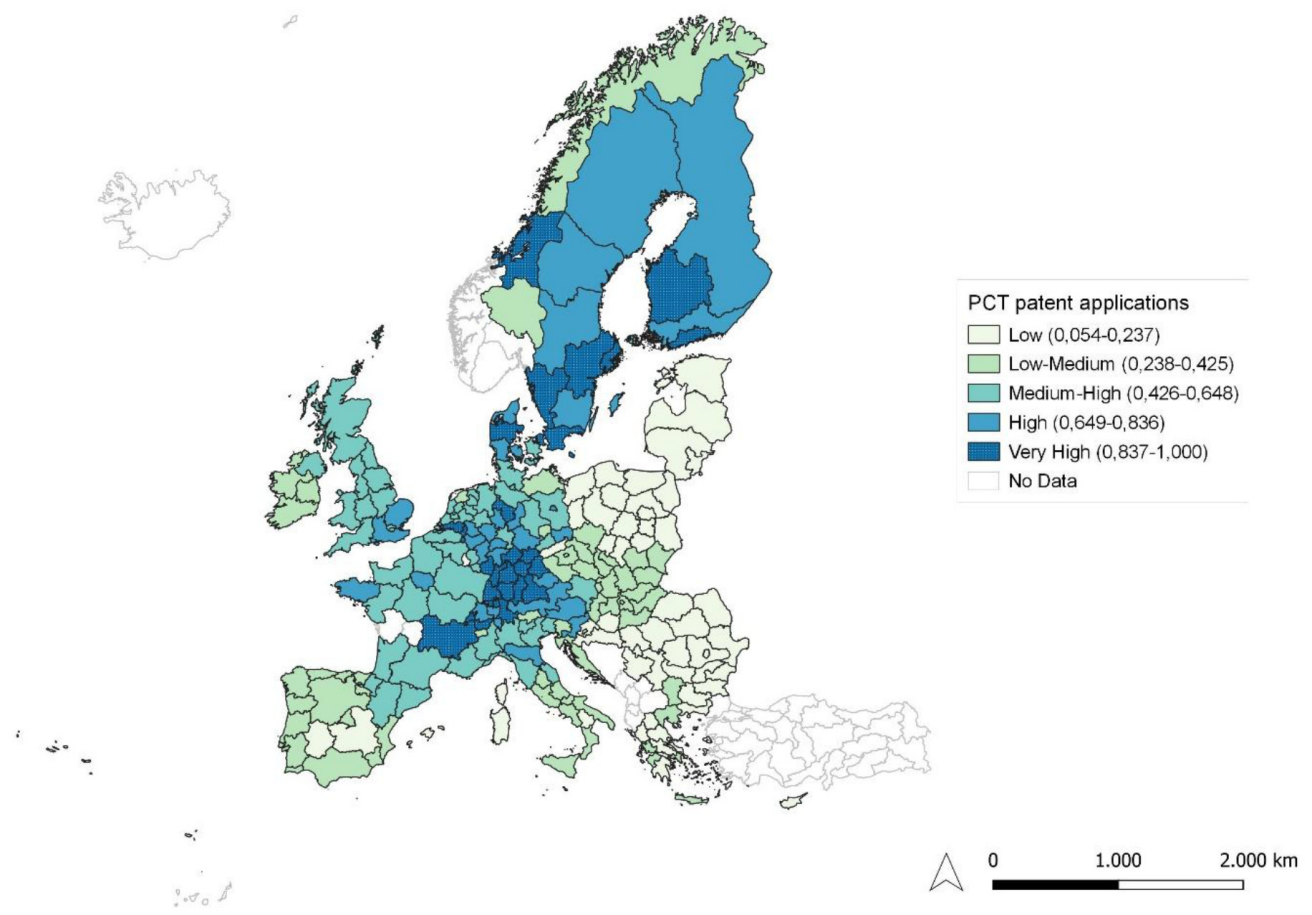
PCT patent applications. Source: elaboration from GISCO – Eurostat data; Data as in Table 1. Map by Silvia Battino.

### 2.6 The role of sales of new-to-market and new-to-firm innovations

Current EU innovation policies are primarily research-oriented, with the main goal of achieving an R&D investment rate of 3.1% of GDP. However, SMEs' innovative activities depend on internal factors, including both R&D and non-R&D factors, as well as external factors such as partnerships with companies and research centers. The recent study of Hervas-Oliver *et al.* (2023) argued that the success of innovation policies depends on a region's potential, as high investments in R&D may not necessarily lead to better performance for SMEs in less developed regions. Consequently, the degree of SMEs' innovation varies among European regions. The findings suggest that SMEs in more developed locations benefit mainly from a combination of all factors, while companies in less innovative regions are heavily reliant on external support.

Due to existing constraints, such as scarcity of resources and capabilities for developing R&D activities ( Hausman, 2005), weak network embeddedness ( Srinivasan *et al.,* 2002), or a lack of highly skilled employees ( Romano, 1990), SMEs are compelled to seek solutions to overcome these barriers. One possible approach is through collaboration. Golonka (2015) posits that a more market-focused cooperation strategy could enhance SMEs' innovativeness. Thus, to minimize regional disparities, more collaborative and location-sensitive policies are required to advance SMEs' innovation activities.

For further investigation, we introduced an indicator that measures the turnover of new or significantly improved products. This includes both new products for the firm and new products for the market. This approach enables the capturing of the creation of cutting-edge technologies (new-to-market products) and the diffusion of these technologies (new-to-firm products).

**Figure 6.**
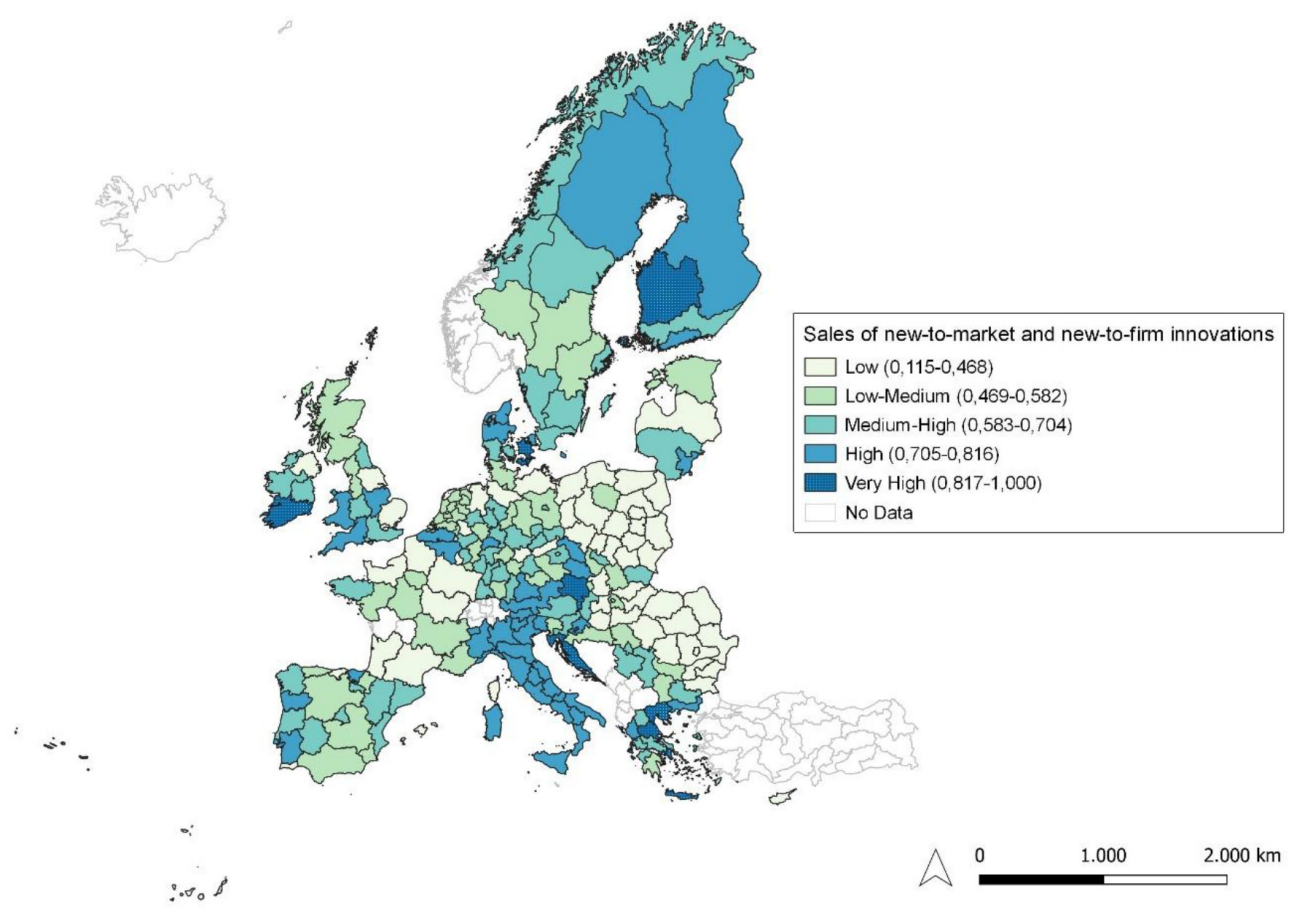
Sales of new-to-market and new-to-firm innovations. Source: elaboration from GISCO – Eurostat data; Data as in Table 1. Map by Silvia Battino.

## 3. Methods

To achieve the objectives of the research, the methodology for the development of the IRE index was executed through a multi-step procedure. After the above-mentioned review of extant literature to identify the critical variables that influence innovation, R&D, and entrepreneurship, with particular attention paid to the role of tertiary education to create the foundational basis for selecting the variables to be included in the IRE index, we focused on the following points:

1. *Analysis of the 2021 "Regional Innovation Scoreboard".* We examined the latest available version of the Regional Innovation Scoreboard (RIS) ( European Commission, 2021) to assess the performance of innovation systems across 240 regions from 22 EU Member States. Additionally, the list of selected countries was expanded to include Norway, Serbia, Switzerland, and the United Kingdom. Cyprus, Estonia, Latvia, Luxembourg, and Malta were also considered, as in these countries, Nomenclature of territorial units for statistics (NUTS) 1 and NUTS 2 levels are identical to the country's territory ( European Commission, 2021).
2. *Selection of ad hoc set of indicators from the "Regional Innovation Scoreboard" 2021*. We selected at least one indicator most closely related to young and student entrepreneurship from the four main types of activities - Framework conditions, Investments, Innovation activities, and Impacts. The six indicators, selected from RIS 2021, are reported in Table 1. It is worth noting that proxies for EIS indicators are included for all enterprises ( European Commission, 2021).

The indicators highlighted in the previous paragraph were related to administrative areas which are generally comparable to the NUTS 2 administrative level. However, they have been adjusted and selected to be homogeneous in terms of the indicators calculated. NUTS 2 level data were mapped particularly for mainland Europe and islands, not considering therefore overseas’ dominions, and including non-EU member states as the UK, Norway, and Switzerland. The IRE indicators, as highlighted in the following sections, as well as GDP and population data, among others, were retrieved and homogenised in time – referred to 2021 – and in currency – data were expressed in Euro.

From EU GISCO databases geographical data were derived and acting as the basis for the computation of the different elaborations. The data consisted of a selection and combination of NUTS 1 and NUTS 2 levels – and similar - administrative units, where ad-hoc indicators were attributed, analysed and mapped. A total of 245 units were used in the analysis. Data regarded EU countries together with the UK and Switzerland.

### 3.1. Creation of the composite index Innovation Readiness Environment (IRE)

We applied the *Mazziotta-Pareto* composite index to summarize the data of the mathematical combination of the selected indicators in order to create the composite index “Innovation Readiness Environment” (IRE) that represents, with a single score, the overall performance measured by the six indicators ( Mazziotta & Pareto, 2013; Mazziotta & Pareto, 2018). We conceived it as a synthesis of all indicators with the purpose of assessing the overall performance (as opposed to a single-indicator performance) of the European Regions.

The MPI can be employed to compare inequality levels across different regions, time periods, or social groups, and it can also be used to evaluate the impact of specific policy interventions on inequality patterns.

The MPI building proceeds in the following two stages:

1) Normalization

Let *X* = *x _ij_* be the matrix with *n* rows (countries or geographical areas) and *m* columns (indicators) and let *M _x _j__* and *S _x _j__* denote the mean and the standard deviation of the *j*-th indicator:

$$M_{x_{j}}=\frac{\sum_{j=1}^{n}x_{ij}}{n}\;S_{x_{j}}=\sqrt{\frac{\sum_{j=1}^{n}{\left(x_{ij}-M_{x_{j}}\right)}^{2}}{n}}.$$

The standardized matrix *Z = z _ij_* is defined as follows:

$$z_{ij}=100\pm \frac{{\left(x_{ij}-M_{x_{j}}\right)}^{2}}{S_{x_{j}}}10$$

where the sign ± depends on the relation of the *j*-th indicators with the phenomenon to be measured (+ if the individual indicator represents a dimension considered positive and - if it represents a dimension considered negative).

2) Aggregation

Let *cv _i_* be the coefficient of variation for the *i*-th units:

$$cv_{i}=\frac{S_{z_{j}}}{M_{z_{j}}}$$

where

$$M_{z_{j}}=\frac{\sum_{j=1}^{n}z_{ij}}{n}\;S_{z_{j}}=\sqrt{\frac{\sum_{j=1}^{n}{\left(z_{ij}-M_{z_{j}}\right)}^{2}}{n}}.$$

Then, the generalized form of MPI is given by:

$$MPI^{\pm }=M_{z_{j}}(1\pm cv_{i}^{2})=M_{z_{i}}\pm S_{z_{i}}cv_{i}$$

where the sign of the penalty (the product *S _z _i__ cv _i_*) depends on the kind of phenomenon to be measured and then on the direction of the individual indicators ( De Muro *et al.*, 2008).

If the indicator is increasing or positive, *i.e.* increasing values of the indicator correspond to positive variations of the phenomenon, then MPI ^–^ is used. Vice versa, if the indicator is decreasing or negative, *i.e.* increasing values of the indicator correspond to negative variation of the phenomenon, then MPI ^+^ is used.

Given our phenomenon the MPI is calculated with the negative sign.

**Figure 7.**
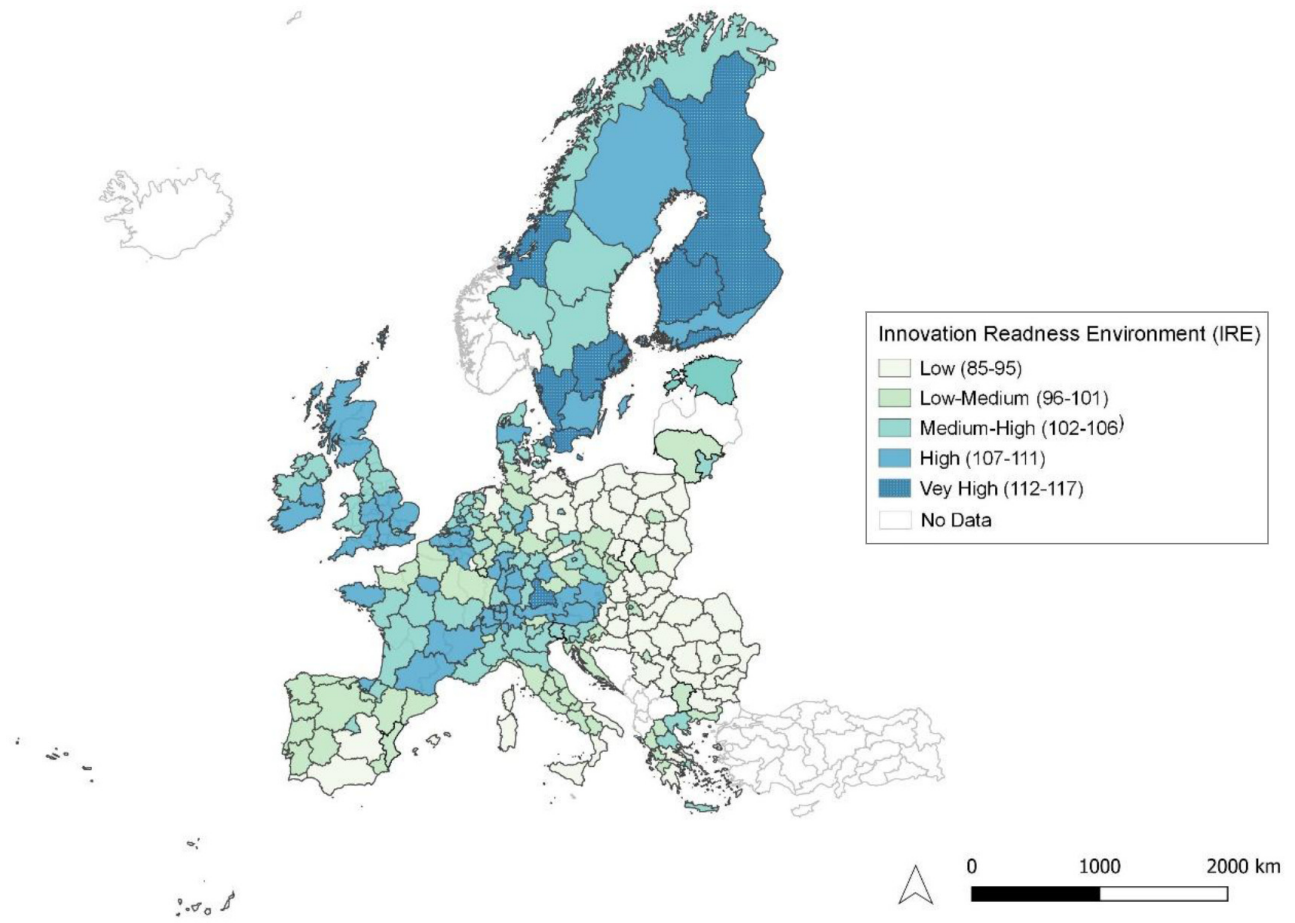
Innovation Readiness Environment. Source: elaboration from GISCO – Eurostat data; Data as in Table 1. Map by Silvia Battino.

Thus, in our IRE index, created using the Mazziotta-Pareto formula shown above, the indicators previously reported in Table 1 co-influence, and with reference to Figure 7, the results show only 11 European countries ranking very high in innovation performance, followed by 44 high-performing countries, 60 (medium-high), 66 (medium-low) and, finally, 64 (low).

### 3.2 An analytical view of IRE as a predictor of innovation

To understand the indicator’s potential, it was decided to perform an analysis of spatial clustering of the same indicator, as well as an exam of a possible relationship between IRE and GDP. This set of analyses involved relying on different tools for performing such tasks, as the linear regression and LISA methods.

***Linear regression*.** A linear regression was performed considering a potential relationship between the IRE and other elements that can be considered relevant, as the GDP. The analysis was performed using Apache OpenOffice 4.1.14 CALC suite A regression analysis was performed over the study area and the spatial units considered. By the linear regression it attempts to model the relationship between the two variables by fitting a linear equation to observed data. The function used calculates the statistics for a line by using the least squares method to calculate a straight line that best fits your data, and then returns an array that describes the line. By using this method, the best-fitting line for the observed data is calculated, minimizing the sum of the squares of the vertical deviations from each data point to the line.

The general equation for the line is:

*Y = aX + b*

Where X is the explanatory variable and Y is the dependent variable. The slope of the line is b, and a is the intercept (the value of y when x = 0).

***Autocorrelation and LISA*.** Being the data considered spatial in their extent, Exploratory Spatial Data Analysis (ESDA) appears of paramount importance in observing the related phenomena in its territorial component. Spatial clustering methods are useful for making sense of complex geographic patterns ( Anselin, 1995). Events in space, in fact, are rarely randomly distributed, but present, instead, a certain degree of local similarity among them. Regions in space, particularly, tend having features locally similar, fading as distance increases or, as Tobler (1970) “all things are related, but nearby things are more related than distant ones”. Data can be, in fact, mutually influencing in terms of geographical shape and spatial proximity and in terms of values attributed to the same units. That means observing the behaviour of a selected variable in reference to its position in space and to its proximity. Such a characteristic is known as spatial autocorrelation, The most interesting property of spatial autocorrelation is the capability to analyse at the same time locational and attribute information ( Goodchild, 1986; Lee & Wong, 2001) defined spatial autocorrelation as follows:

$$SAC=\frac{\sum_{i=1}^{n}\sum_{j=1}^{n}c_{ij}w_{ij}}{\sum_{i=1}^{n}\sum_{j=1}^{n}w_{ij}}$$

Where:

1. *n* is the number of objects;
2. *i* and *j* are two objects;
3. *x _i_* is the value of object *i* attribute;
4. *c _ij_* is a degree of similarity of attributes *i* and *j*;
5. *w _ij_* is a degree of similarity of location *i* and *j*;

if *c _ij_* = ( *x _i_ – x*) ( *x _j_ – x*) Moran Index I ( Moran, 1948; O’Sullivan & Unwin, 2010) can be defined as follows:

$$I=\frac{N\sum_{i}\sum_{j}w_{ij}(x_{i}-\bar{x})(x_{j}-\bar{x})}{(\sum_{i}\sum_{j}w_{ij})\sum_{i}{\left(x_{i}-\bar{x}\right)}^{2}}$$

Moran I - together with Geary’s G, not considered here – represent a global indicator of spatial autocorrelation, considered on the overall study region. Local variations are better observed by means of Local Indicators of Spatial Association, in particular, the Local Moran Index makes it possible to evaluate for each position the similarity of each observation with nearby geographical objects. This can be seen as the sum of all local indices and is proportional to the value of the Moran one:

$$I_{i}=\frac{\left(X_{i}-\bar{X}\right)}{S_{x}^{2}}\sum_{j=1}^{N}\left(w_{ij}(X_{j}-\bar{X})\right)$$

Where:

- N is the number of geographical units;
- X _i_ is the variable describing the phenomenon under investigation in region i;
-
*X* represents the sample average and (X _i_ – X) it is the variable’s average deviation;
-
${\text{S}}_{\text{x}}^{\text{2}}$ is the Standard deviation;
- w _ij_ is the weight matrix.

The area units considered need to be weighted by means of a contiguity matrix, reproducing the spatial relationship among the regions considered, using a binary set of existing / non existing contiguity among the different areas, following a ‘Queen rule’ of connection ( Anselin, 1995).

From the application of the above mentioned method it is possible to obtain five combinations:

(1) high values of the phenomenon and high levels of similarity with the nearby areas, known hot spots (High-High), observable on the upper right quadrant of the global Moran’s I graph;
(2) low values of the phenomenon and low levels of similarity with the nearby areas, called cold spots (Low-Low); observable on the lower left quadrant of the global Moran’s I graph
(3) high values of the phenomenon and low levels of similarity with the nearby areas are detected, referred to as potential outliers (High-Low);
(4) low values of the phenomenon and high levels of similarity with nearby areas are highlighted, referred to as potential outliers (Low-High);
(5) no significant autocorrelation values are detected (Not Significant).

Spatial autocorrelation can be also considered as a bivariate one, typically as the correlation between one variable and the spatial lag of another variable. In the case considered, the implemented indicator, IRE, was related to measures of local GDP in order to understand the existence of a possible relationship among the different variable and their spatial extent. Recently LISA’s local Moran’s I was used to analyse, among others, socio economic phenomena on area unit data on various applications, from migration movements ( Borruso, 2009; Murgante & Borruso, 2012) to the efficacy of cohesion policies ( Balletto *et al.,* 2020). The analysis was performed using GeoDA as Free and Open Source Software, as developed by Anselin (1995).

## 4. Results

**Figure 8.**
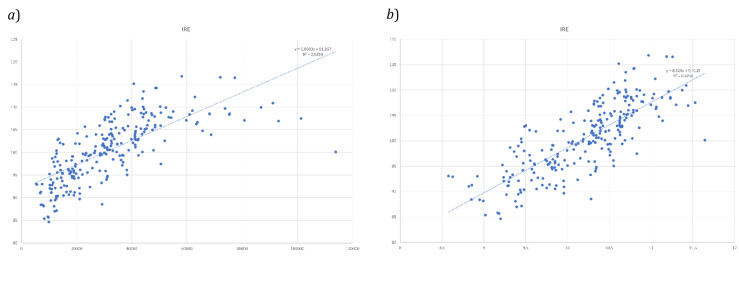
Regression Analysis **a**) IRE and GDP; **b**) IRE – Ln GDP. Source: elaboration from GISCO – Eurostat data; Data as in Table 1. Graph by Giuseppe Borruso.

IRE applied to the 245 European NUTS 1 and NUTS 2 units considered was related to per capita GDP values in € at 2021 and a set of analyses were performed, as anticipated in the methods above. Namely, the regression analysis and the Local Moran over the IRE index. As an early, visual observation of the IRE maps realized portrayed a certain level of similarity of most innovative regions with the highest GDP’s ones, as those of the European core or the heart of the so-called ‘Blue Banana’ ( Brunet, 1989), a regression analysis was initially carried out to observe if a certain relationship among the variables could be considered. IRE and GDP values were plotted, and a linear regression performed, providing interesting results given the high level of relation that can be observed among the different variables (R ^2^ of 0.525; Figure 8 a) in the direct relationship, and better fitting values considering Ln GDP (R ^2^ of 0.6256; Figure 8 b).

**Figure 9.**
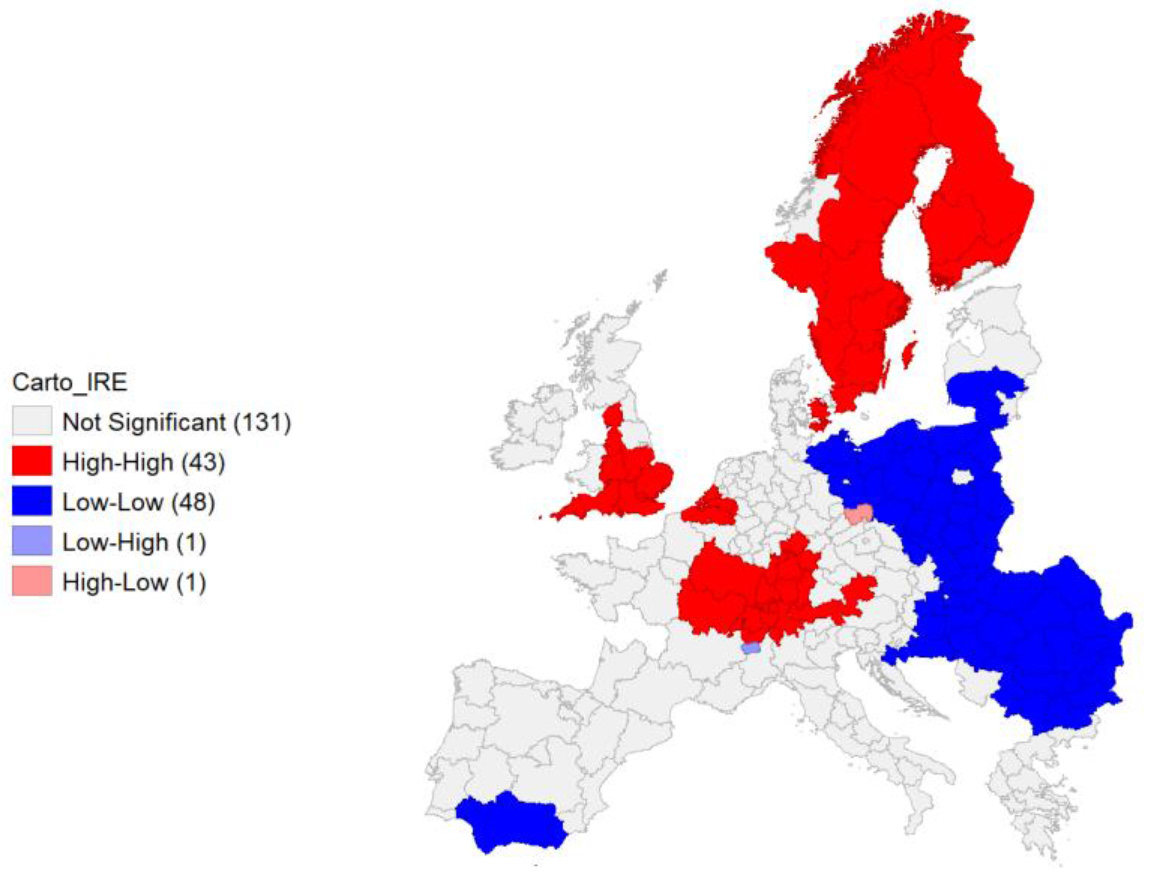
Local Moran’s I Cluster Map on IRE. Source: elaboration from GISCO – Eurostat data; Data as in Table 1. Map by Giuseppe Borruso.

**Figure 10.**
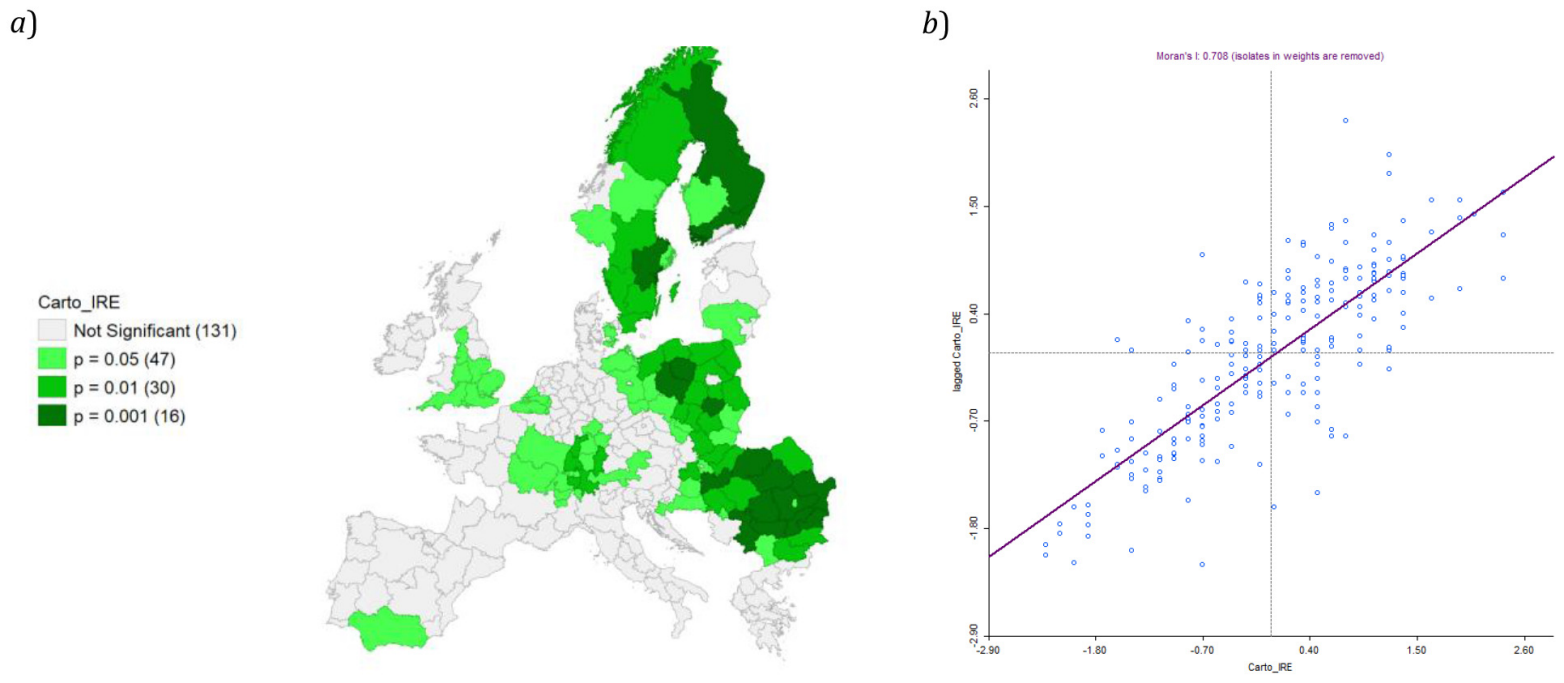
***a***) Local Moran’s I Significance map on IRE and ***b***) Moran’s I. Source: elaboration from GISCO – Eurostat data; Data as in Table 1. Map and graph by Giuseppe Borruso.

**Figure 11.**
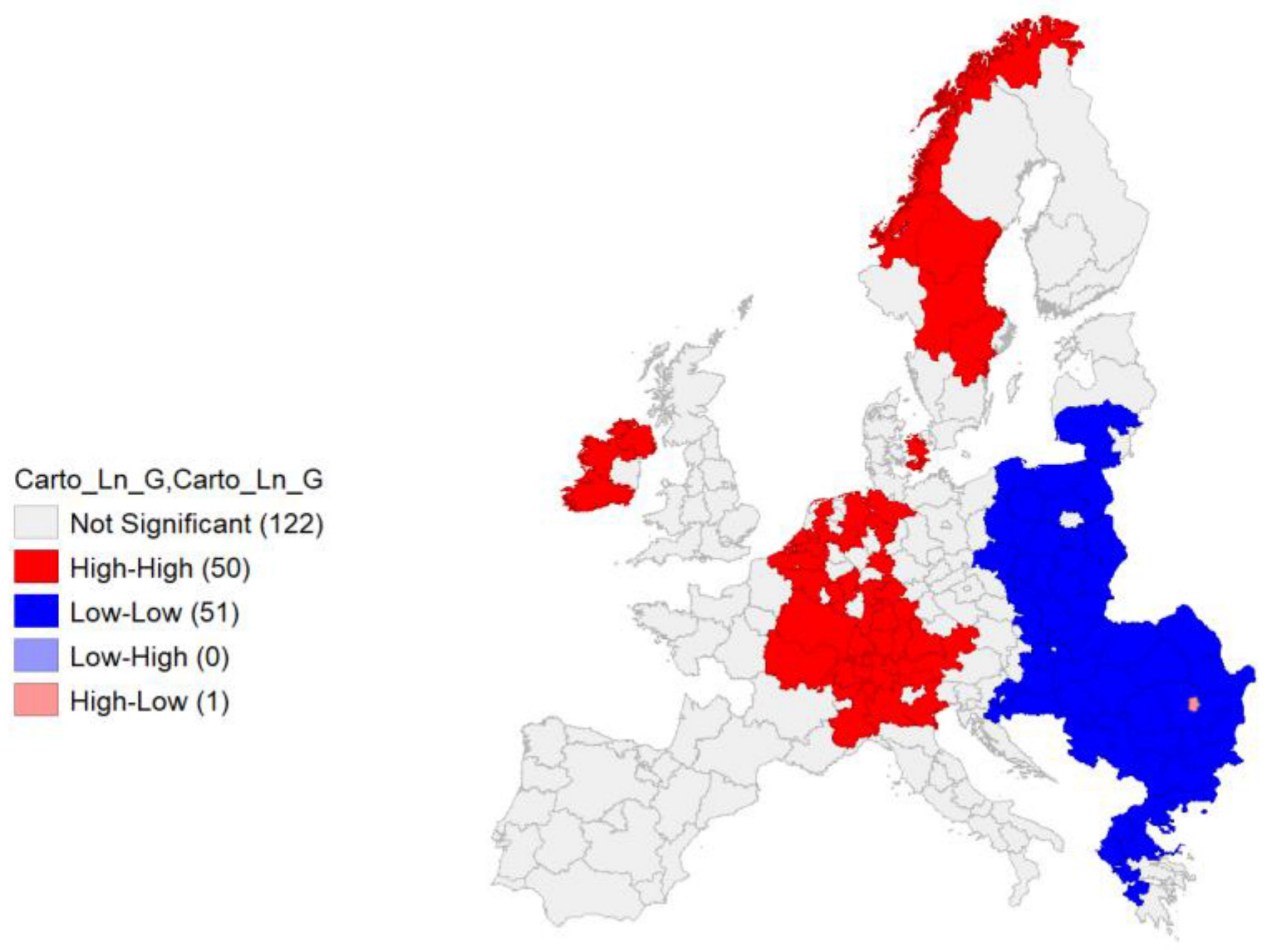
Local Moran’s I Cluster Map on Ln GDP. Source: elaboration from GISCO – Eurostat data; Data as in Table 1. Map by Giuseppe Borruso.

**Figure 12.**
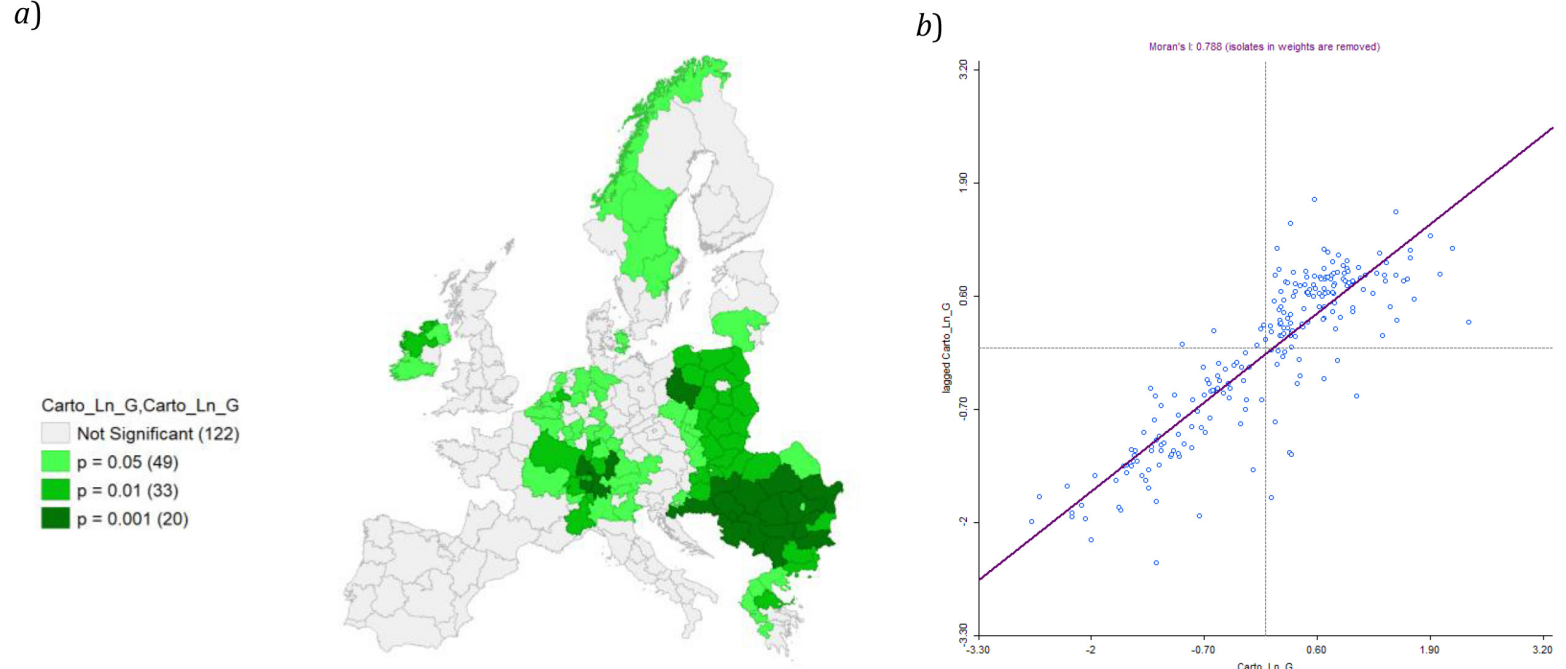
*a*) Local Moran’s I Significance map on Ln GDP and *b*) Moran’s I. Source: elaboration from GISCO – Eurostat data; Data as in Table 1. Map and graph by Giuseppe Borruso.

**Figure 13.**
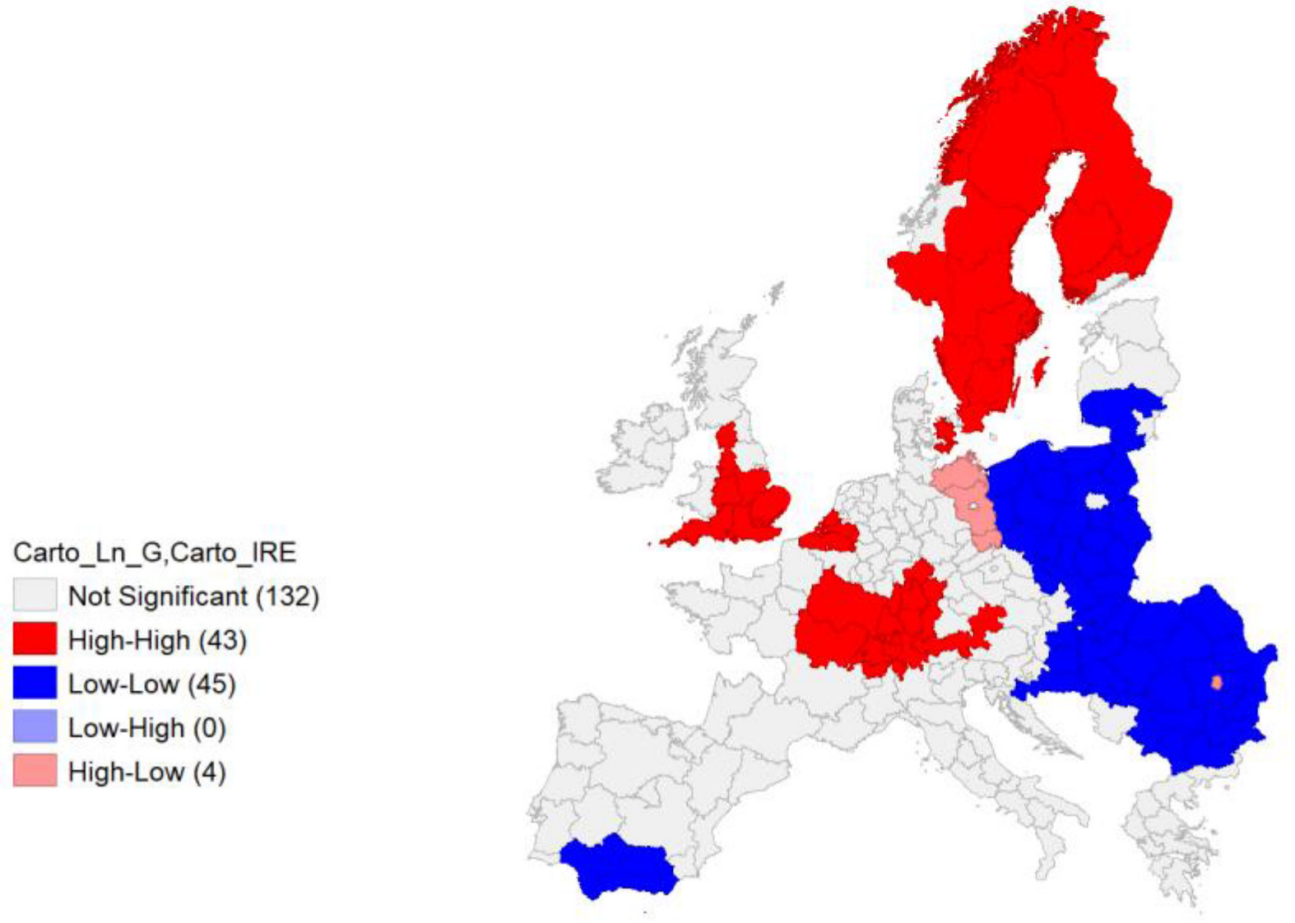
Bivariate Local Moran’s I Cluster Map on IRE & Ln GDP. Source: elaboration from GISCO – Eurostat data; Data as in Table 1. Map by Giuseppe Borruso.

**Figure 14.**
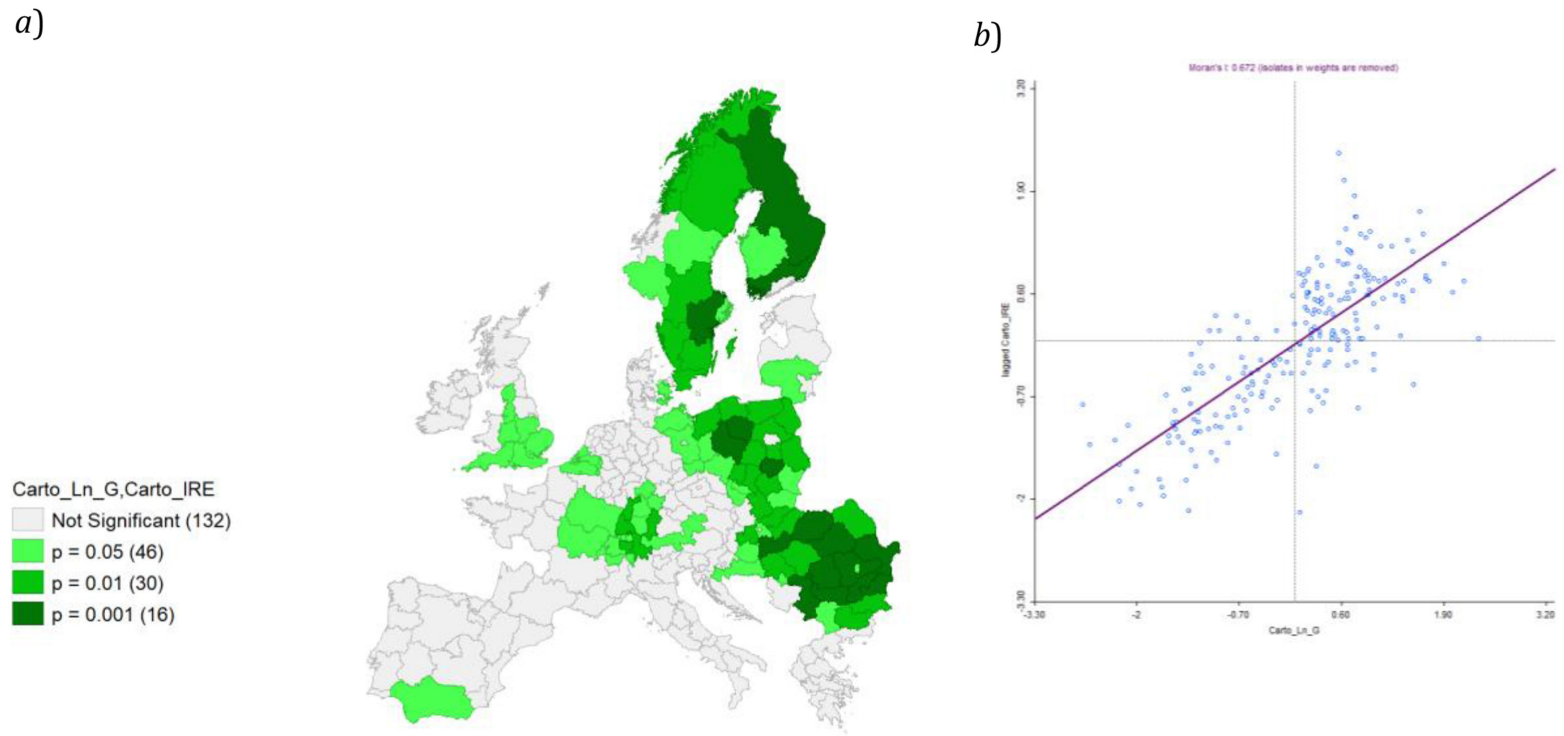
*a*) Bivariate Local Moran’s I Significance map on IRE & Ln GDP and *b*) Moran’s I. Source: elaboration from GISCO – Eurostat data; Data as in Table 1. Map and graph by Giuseppe Borruso.

Such initial results induced us to consider the spatial component and behaviour of both IRE and of its relationship with European GDP. As in previous paragraph, Global and Local Moran’s I were performed on IRE ( Figure 9 and Figure 10), Ln GDP ( Figure 11 and Figure 12) and as a crossed, bivariate Moran’s I on IRE and Ln GDP ( Figure 13 and Figure 14).

Local Moran’s application present spatial clusters of high-high autocorrelation of the IRE in the NUTS areas in proximity of the European core, covering from Eastern France, Southern Germany, part of Austria and Belgium, as well as most of England units. Scandinavian peninsula’s units represent a relevant cluster as well. Negative autocorrelation can be observed on a wide cluster of units belonging to Eastern European countries, as well as on one in Southern Spain ( Figure 9).

Cluster map appears significant, with a relevant value of Moran’s I (0,708; Figure 10 a and b).

Cluster map appears significant, with a relevant value of Moran’s I (0,708; Figure 10 a and b).

Univariate local Moran’s I was performed also on Ln GDP, reporting a – quite expectable – autocorrelation and spatial clustering of areas in the European economic core, rooted into Northern Italian Regions and extended northwards through Southern and Western Germany, Eastern France, BeNeLux and Ireland. Scandinavian regions represent another clusters. A East-West division appears with the wide cluster of negative autocorrelation regarding the regions belonging to Eastern European countries ( Figure 11).

Also in this case cluster map appears significant, with a relevant value of Moran’s I (0,788; Figure 12 a and b).

Also in this case cluster map appears significant, with a relevant value of Moran’s I (0,788; Figure 12 a and b).

A final analysis was performed by means of a bivariate local Moran’s I, comparing IRE and Ln GDP ( Figure 13). As bivariate local Moran's I describes the statistical relationship between the first variable at a given location and the spatially lagged second variable at neighbouring locations, the ‘high – high’ values (red) represent the spatial autocorrelation of the area units (also observable in the upper right quadrant of the Moran’s I graph, Figure 14 b), characterized by high values of IRE, spatial contiguity, and high values of Ln GDP. An interesting picture can be observed, with clusters of NUTS areas in the Central and Southern part of the European core – Southwestern Germany, Western France, Southern Austria – Belgian NUTS and most of England Units. Scandinavian NUTS represent a relevant cluster as well. Negative spatial autocorrelation covers a relevant part of Eastern European Countries’ NUTS.

The results from the bivariate local Moran’s I computation can be considered significant ( Figure 14 a and b) with a relevant value of Global Moran’s I (0.672).

## 5. Conclusions

In the evolving discourse on the spatial dynamics of innovation clusters within European regions, it is crucial to highlight the critical role of the newly developed Index of Regional Entrepreneurship (IRE) in outlining the intricate patterns and associations with per capita GDP distributions.

If it is true that a European core tend to be quite settled and consolidated as in the Brunet (1989) Blue Banana metaphor, innovation tend to follow partly similar patterns but also other, more specific ones. Focusing on the British Isles and the Scandinavian peninsula, the IRE index serves as an invaluable tool for nuanced exploration of different regional policy frameworks, revealing institutional and structural dimensions beyond GDP narratives.

Weaving this analysis into the current literature requires an alignment with the objectives of Entrepreneurship 2020. The strategy is based on three pillars, all of which reflect on the IRE index: a robust framework for entrepreneurial education and training, the promotion of an environment ripe for entrepreneurial growth, and the cultivation of role models to unleash the latent entrepreneurial capacity within different social groups.

The need to strengthen entrepreneurial education and training is central to this strategy, representing a key investment to enhance Europe's position in the global entrepreneurial landscape. In this context, the IRE Index emerges as a cornerstone that allows for a deeper understanding of the region-specific nuances that influence entrepreneurial developments, as outlined in the writings of Jenner (2012) and in the guidelines that are encapsulated within the DG EAC - ET2020.

However, Benneworth and Osborne (2015) argued that the untapped potential in entrepreneurship education is largely due to inadequate integration within university knowledge frameworks. Thus, the IRE Index can act as an analytical tool, guiding research efforts to synergize entrepreneurial initiatives with university knowledge, fostering the ecosystem where entrepreneurial attitudes can be developed. Simultaneously, the rapidly evolving global knowledge and technology landscape highlighted by Posselt *et al.* (2019) requires a proactive stance in advancing entrepreneurial universities capable of navigating this complexity. IRE Index, in this regard, is emerging as a tool to guide universities in fostering leadership and innovation, thus, realising Entrepreneurship 2020 Action Plan's vision that supports sustainable economic growth and development in a coherent and inclusive way.

Synthesising these findings within the broader framework of the 2030 Agenda for Sustainable Development (SDG), the IRE Index is seen as a central tool for improving societal wellbeing, particularly for disadvantaged groups. The relationships between entrepreneurship and the SDG, in particular Goals 4.4 and 8.3, comes alive through the lens of the IRE Index, providing critical evidence on the role of entrepreneurship in fostering skills development and innovation, an, as a result, inclusive growth. Besides, promoting entrepreneurship, prosperity and well-being across Europe requires taking into account regional differences and a deeper understanding of regional innovation indicators. Using tools such as the Smart Specialisation Strategy (S3) and the Smart Specialisation Strategies for Sustainable and Inclusive Growth (S4), the IRE Index provides an in-depth understanding of regional entrepreneurial trends to guide future research and policy directions.

## Ethics and consent

Ethical approval and consent were not required.

## Data Availability

The dataset is available at the following link: Figshare: EIS Data 2021. https://doi.org/10.6084/m9.figshare.24459265.v2 ( Fabbro *et al*., 2023) The dataset encompasses the year 2021 from European Innovation Scoreboard (EIS) 2021 Database and focuses on European Union countries, specifically addressing the following indicators: 1.1.2, 1.1.3, 2.2.1, 3.2.1, 3.3.1, 4.2.3. Data are available under the terms of the Creative Commons Zero “No rights reserved” data waiver (CC0 1.0 Public domain dedication). Free and Open Source Software was used for realizing maps (QGIS 3.24, https://www.qgis.org/), linear regression (CALC from Apache OpenOffice 4.1.14, https://www.openoffice.org/), Local Indicator of Spatial Association (GeoDA 1.22, https://geodacenter.github.io/). The data presented herein are extracted from the European Innovation Scoreboard (EIS) 2021 Database, which is publicly accessible at the following URL: https://ec.europa.eu/research-and-innovation/en/statistics/performance-indicators/european-innovation-scoreboard/eis Documentation pertaining to the dataset is accessible via the following URL: https://research-and-innovation.ec.europa.eu/statistics/performance-indicators/european-innovation-scoreboard_en
