# Supplementary material for: Is entrepreneurship a key factor in the development of European countries? A proposal for an innovation readiness environment (IRE) index
Source: Open Res Eur. 2024 Jun 24;4:12. Originally published 2024 Jan 8. [Version 2] doi: 10.12688/openreseurope.16813.2 (PMC11024594; doi:10.12688/openreseurope.16813.2)
Supplement: Peer review history

# Reviewer report: Reviewer response for version 2

**DOI:** 10.21956/openreseurope.19272.r41609

**Published:** 2024-07-10

**Authors:** Maja Basic

**Affiliations:** 1 Faculty of Economics and Business, University of Zagreb, Zagreb, Croatia

The authors revised the paper according to the both reviewers' comments and there are no more suggestions.

If applicable, is the statistical analysis and its interpretation appropriate?

Yes

Is the study design appropriate and is the work technically sound?

Yes

Is the work clearly and accurately presented and does it engage with the current literature?

Yes

Are the conclusions drawn adequately supported by the results?

Yes

Are sufficient details of methods and analysis provided to allow replication by others?

Yes

Are all the source data and materials underlying the results available?

Yes

Reviewer Expertise:

innovation, internationalisation, competitiveness

I confirm that I have read this submission and believe that I have an appropriate level of expertise to confirm that it is of an acceptable scientific standard.

# Reviewer report: Reviewer response for version 1

**DOI:** 10.21956/openreseurope.18166.r37926

**Published:** 2024-04-17

**Authors:** Maja Basic

**Affiliations:** 1 Faculty of Economics and Business, University of Zagreb, Zagreb, Croatia

Thank you for the opportunity to read and evaluate your works. It is an interesting study that speaks about the relationship between innovation, R&D, and entrepreneurship in European nations, emphasizing the role of tertiary educational institutions. It introduces the Innovation Readiness Environment (IRE) index to evaluate regional innovative capabilities, highlighting the importance of higher education in fostering entrepreneurial success and suggesting targeted policy interventions.

 The IRE Index is a novelty and an interestingly presented index. Attempts have been made to compose the index, especially in organisations and not so much in locally or regionally (apart from the mentioned Regional Innovation Scoreboard). Hence, this index presents a valuable novelty to the research on entrepreneurship and innovation. 

 Moreover, it soundly connects the concepts of the index to the SDGs and the Entrepreneurship 2020 Action Plan. 

 The methodology is sound and accounts for a new approach to measuring the innovation activities. 

 The only minor drawback is incorporation of aspects of the Regional Innovation Indicators without further attempt to include different perspective such as the usage of GERD, especially significant in the former socialist countries, and furthering this index into different categories. This is a suggestion for further research. E.g., by using the study of Yoruk, Radosevic and Fisher (2023) or Radosevic and Soete (2023) to gain additional insights and connect the technology trajectory to entrepreneurship.

If applicable, is the statistical analysis and its interpretation appropriate?

Yes

Is the study design appropriate and is the work technically sound?

Yes

Is the work clearly and accurately presented and does it engage with the current literature?

Yes

Are the conclusions drawn adequately supported by the results?

Yes

Are sufficient details of methods and analysis provided to allow replication by others?

Yes

Are all the source data and materials underlying the results available?

Yes

Reviewer Expertise:

innovation, internationalisation, competitiveness

I confirm that I have read this submission and believe that I have an appropriate level of expertise to confirm that it is of an acceptable scientific standard.

# Response

**Published:** 2024-06-11

**Authors:** Giacomo Marzi

**Affiliations:** IMT School for Advanced Studies Lucca, Lucca, Tuscany, Italy

***Comment 1:*** Thank you for the opportunity to read and evaluate your works. It is an interesting study that speaks about the relationship between innovation, R&D, and entrepreneurship in European nations, emphasizing the role of tertiary educational institutions. It introduces the Innovation Readiness Environment (IRE) index to evaluate regional innovative capabilities, highlighting the importance of higher education in fostering entrepreneurial success and suggesting targeted policy interventions.

 The IRE Index is a novelty and an interestingly presented index. Attempts have been made to compose the index, especially in organisations and not so much in locally or regionally (apart from the mentioned Regional Innovation Scoreboard). Hence, this index presents a valuable novelty to the research on entrepreneurship and innovation. 

 Moreover, it soundly connects the concepts of the index to the SDGs and the Entrepreneurship 2020 Action Plan. 

 The methodology is sound and accounts for a new approach to measuring the innovation activities. 

 The only minor drawback is incorporation of aspects of the Regional Innovation Indicators without further attempt to include different perspective such as the usage of GERD, especially significant in the former socialist countries, and furthering this index into different categories. This is a suggestion for further research. E.g., by using the study of Yoruk, Radosevic and Fisher (2023) or Radosevic and Soete (2023) to gain additional insights and connect the technology trajectory to entrepreneurship.  

***Response: *** Thank you for your thoughtful review of our study. We value your feedback and constructive suggestions for future research. Your insights have been carefully considered and integrated into the section on limitations and future research directions. Your contribution has greatly enriched our work, and we are grateful for your valuable input.

# Reviewer report: Reviewer response for version 1

**DOI:** 10.21956/openreseurope.18166.r37933

**Published:** 2024-02-20

**Authors:** Leonardo Boncinelli

**Affiliations:** 1 University of Florence, Florence, Italy

**Summary**

 This study makes a significant contribution to understanding the interplay between innovation, R&D, and entrepreneurship across European regions. The introduction of the IRE index is a noteworthy advancement in measuring regional innovation capabilities. 

**Suggestions**

 The manuscript would be enhanced by a more thorough comparative analysis with the existing body of literature. Additionally, it would benefit from an expanded exposition on the criteria employed for the selection of indicators. Establishing a more robust linkage between the constructed index and its implications for policy-making and economic growth, while concurrently acknowledging its present constraints, would markedly augment the contribution of this paper. Addressing these aspects would not only sharpen the study's impact but also amplify its significance

**A. Clarity and Engagement with Current Literature**

 Answer: Yes/Partly

 The abstract and introduction indicate a clear attempt to engage with the current literature on innovation, R&D, and entrepreneurship. The inclusion of a novel metric, the Innovation Readiness Environment (IRE) index, suggests an effort to contribute to the existing body of knowledge. However, the depth of engagement with current literature could be further elaborated upon to demonstrate how this work builds upon or diverges from existing research findings.

*Recommendations*:

 - Enhance the literature review section by comparing and contrasting the IRE index with other existing metrics in innovation studies (e.g., GII & EIS).

 - Sketch out (verbally) a theoretical framework that interconnects the various components of the IRE index, to provide a more comprehensive understanding of the overarching context.

**B. Study Design and Technical Soundness**

 Answer: Yes

 The study design, focusing on the development and application of the IRE index across European nations, seems appropriate for addressing the research objectives. The methodology section describes a thorough process for selecting variables, creating the composite index, and analyzing the data, which supports the work's technical soundness.

**C. Details of Methods and Analysis for Replication**

 Answer: Yes/Partly

 While the methodology for creating the IRE index and conducting analyses is detailed, the description could benefit from more specific information on the selection of the indicators.

*Recommendation*:

 - Expand upon the descriptions of the data sources utilized in the study, delineating any limitations or potential biases associated with these sources; furthermore, elucidate the rationale behind the selection of the specific indicators included in the IRE index.

**D. Availability of Source Data and Materials**

 Answer: Yes

 The article provides a DOI for accessing the dataset. This transparency in data availability is commendable and supports the reproducibility of the research.

**E. Statistical Analysis and Interpretation**

 Answer: Yes/Partly

 The study employs appropriate statistical analyses, including linear regression and spatial clustering methods. However, the interpretation of these analyses could be enhanced by discussing the implications of the findings in a broader economic and societal context.

*Recommendation*:

 - Discuss the statistical significance of the findings and their practical implications for policymakers and stakeholders in the innovation ecosystem.

**F. Support of Conclusions by Results**

 Answer: Yes/Partly

 The conclusions drawn about the importance of tertiary education and the regional specificity of innovation activities are supported by the results. However, the direct impact of the IRE index on policy formulation and economic growth could be further substantiated with empirical examples or case studies.

*Recommendation*:

 - Discuss any limitations of the study and how future research could address these gaps.

If applicable, is the statistical analysis and its interpretation appropriate?

Yes

Is the study design appropriate and is the work technically sound?

Yes

Is the work clearly and accurately presented and does it engage with the current literature?

Yes

Are the conclusions drawn adequately supported by the results?

Yes

Are sufficient details of methods and analysis provided to allow replication by others?

Yes

Are all the source data and materials underlying the results available?

Yes

Reviewer Expertise:

Behavioral economics; Experimental economics; Game theory

I confirm that I have read this submission and believe that I have an appropriate level of expertise to confirm that it is of an acceptable scientific standard.

# Response

**Published:** 2024-06-11

**Authors:** Giacomo Marzi

**Affiliations:** IMT School for Advanced Studies Lucca, Lucca, Tuscany, Italy

***Comment 1:*** **Summary**

 This study makes a significant contribution to understanding the interplay between innovation, R&D, and entrepreneurship across European regions. The introduction of the IRE index is a noteworthy advancement in measuring regional innovation capabilities. 

***Response: *** We believe that the IRE Index provides valuable insights for policymakers and stakeholders to facilitate more effective promotion of innovation and entrepreneurial activities. Your encouraging comments motivate us to continue refining and expanding our research to increase its impact and practical applications in this area.  

***Comment 2:***

**Suggestions**

 The manuscript would be enhanced by a more thorough comparative analysis with the existing body of literature. Additionally, it would benefit from an expanded exposition on the criteria employed for the selection of indicators. Establishing a more robust linkage between the constructed index and its implications for policy-making and economic growth, while concurrently acknowledging its present constraints, would markedly augment the contribution of this paper. Addressing these aspects would not only sharpen the study's impact but also amplify its significance.  

***Response: *** In response, we have extended our literature review to include a more thorough comparative analysis with existing metrics such as the Global Innovation Index (GII), European Innovation Scoreboard (EIS), Regional Innovation Scoreboard (RIS), Global Entrepreneurship Index (GEI), OECD Science, Technology and Industry Scoreboard. We have also provided a more detailed explanation of the criteria used to select the indicators and clarified the rationale for their inclusion. In addition, we have provided a more robust link between the IRE index and its implications for policymaking and economic growth. We have acknowledged the current limitations of the index and suggested future research directions to address these gaps. These improvements have increased the impact and relevance of the study.

***Comment 3:***

**A. Clarity and Engagement with Current Literature**

 Answer: Yes/Partly

 The abstract and introduction indicate a clear attempt to engage with the current literature on innovation, R&D, and entrepreneurship. The inclusion of a novel metric, the Innovation Readiness Environment (IRE) index, suggests an effort to contribute to the existing body of knowledge. However, the depth of engagement with current literature could be further elaborated upon to demonstrate how this work builds upon or diverges from existing research findings.

*Recommendations*:

 - Enhance the literature review section by comparing and contrasting the IRE index with other existing metrics in innovation studies (e.g., GII & EIS).

 - Sketch out (verbally) a theoretical framework that interconnects the various components of the IRE index, to provide a more comprehensive understanding of the overarching context. ***Response: *** We have taken on board your recommendations to improve clarity and engagement with the current literature. In particular, we have expanded the literature review section to include a brief overview of other existing metrics in innovation studies, such as the Global Innovation Index (GII), the European Innovation Scoreboard (EIS), the Regional Innovation Scoreboard (RIS), the Global Entrepreneurship Index (GEI), and the OECD Science, Technology and Industry Scoreboard. To provide a more comprehensive understanding of the overarching context, we have also outlined a theoretical framework that links the different components of the IRE index.

***Comment 4:***

**B. Study Design and Technical Soundness**

 Answer: Yes

 The study design, focusing on the development and application of the IRE index across European nations, seems appropriate for addressing the research objectives. The methodology section describes a thorough process for selecting variables, creating the composite index, and analyzing the data, which supports the work's technical soundness. ***Response: *** We appreciate your acknowledgment of the appropriateness of the study design and the thoroughness of the methodology section. Our aim was to develop and apply the IRE index effectively across European countries, and we are pleased that you found our approach appropriate for achieving the research objectives. Thank you for recognising these aspects of our study.

***Comment 5:***

**C. Details of Methods and Analysis for Replication**

 Answer: Yes/Partly

 While the methodology for creating the IRE index and conducting analyses is detailed, the description could benefit from more specific information on the selection of the indicators.

*Recommendation*:

 - Expand upon the descriptions of the data sources utilized in the study, delineating any limitations or potential biases associated with these sources; furthermore, elucidate the rationale behind the selection of the specific indicators included in the IRE index. ***Response: *** We have taken steps to address your suggestion by enhancing the methodology section to provide more specific information on indicator selection.

***Comment 6:***

**D. Availability of Source Data and Materials**

 Answer: Yes

 The article provides a DOI for accessing the dataset. This transparency in data availability is commendable and supports the reproducibility of the research. ***Response: *** We appreciate your recognition of the transparency of data availability demonstrated in our article. We are committed to maintaining this standard of transparency to facilitate replication and verification of our findings.

***Comment 7:***

**E. Statistical Analysis and Interpretation**

 Answer: Yes/Partly

 The study employs appropriate statistical analyses, including linear regression and spatial clustering methods. However, the interpretation of these analyses could be enhanced by discussing the implications of the findings in a broader economic and societal context.

*Recommendation*:

 - Discuss the statistical significance of the findings and their practical implications for policymakers and stakeholders in the innovation ecosystem. ***Response: *** Thank you for your feedback. We acknowledge that although our study makes use of appropriate statistical analyses, there is still room for improvement in the interpretation of these analyses. Following your recommendation, we have added a discussion of the statistical significance of the findings and their practical implications for policymakers and innovation ecosystem stakeholders in the Conclusions section. ***Comment 8:***

**F. Support of Conclusions by Results**

 Answer: Yes/Partly

 The conclusions drawn about the importance of tertiary education and the regional specificity of innovation activities are supported by the results. However, the direct impact of the IRE index on policy formulation and economic growth could be further substantiated with empirical examples or case studies.

*Recommendation*:

 - Discuss any limitations of the study and how future research could address these gaps. ***Response: *** Thank you for your comments! We appreciate your insightful input, which contributes to a deeper understanding of the implications of our study. We have addressed the limitations of the study and provided insights into how future research could fill these gaps in response to your recommendations.
